# Author Correction: Identification of markers for neurescence through transcriptomic profiling of postmortem human brains

**DOI:** 10.1038/s41514-025-00262-9

**Published:** 2025-08-12

**Authors:** Shiva Kazempour Dehkordi, Sogand Sajedi, Amirreza Heshmat, Miranda E. Orr, Habil Zare

**Affiliations:** 1 Glenn Biggs Institute for Alzheimer’s and Neurodegenerative Diseases, San Antonio, TX USA; 2 Department of Cell Systems and Anatomy, University of Texas Health San Antonio, San Antonio, TX USA; 3 Leukemia Department, The University of Texas MD Anderson Cancer Center, Houston, TX USA; 4 Department of Imaging Physics, the University of Texas MD Anderson Cancer Center, Houston, TX USA; 5 Department of Neurology, Washington University School of Medicine, St. Louis, MO USA; 6 US Department of Veterans Affairs, Washington, WA USA

**Keywords:** Senescence, Neurological disorders, Dementia, Alzheimer's disease, Biomarkers

Correction to: *npj Aging* 10.1038/s41514-025-00235-y, published online 1 July 2025

In the Author Contributions section of this article, the contribution for S.S. was incomplete. The original text stated that S.S. “conducted differential expression and pathway enrichment analyses, prepared Figs. 2–4.” This should have read: S.S. “developed code, conducted differential expression and pathway enrichment analyses, prepared Figs. 2–4.” The original article has been corrected.

