# Supplementary material for: Identification of markers for neurescence through transcriptomic profiling of postmortem human brains
Source: NPJ Aging. 2025 Jul 1;11(1):57. doi: 10.1038/s41514-025-00235-y (PMC12214559; doi:10.1038/s41514-025-00235-y)
Supplement: Supplementary file 1 — Supplementary figures [file 41514_2025_235_MOESM1_ESM.pdf]

Supplementary material

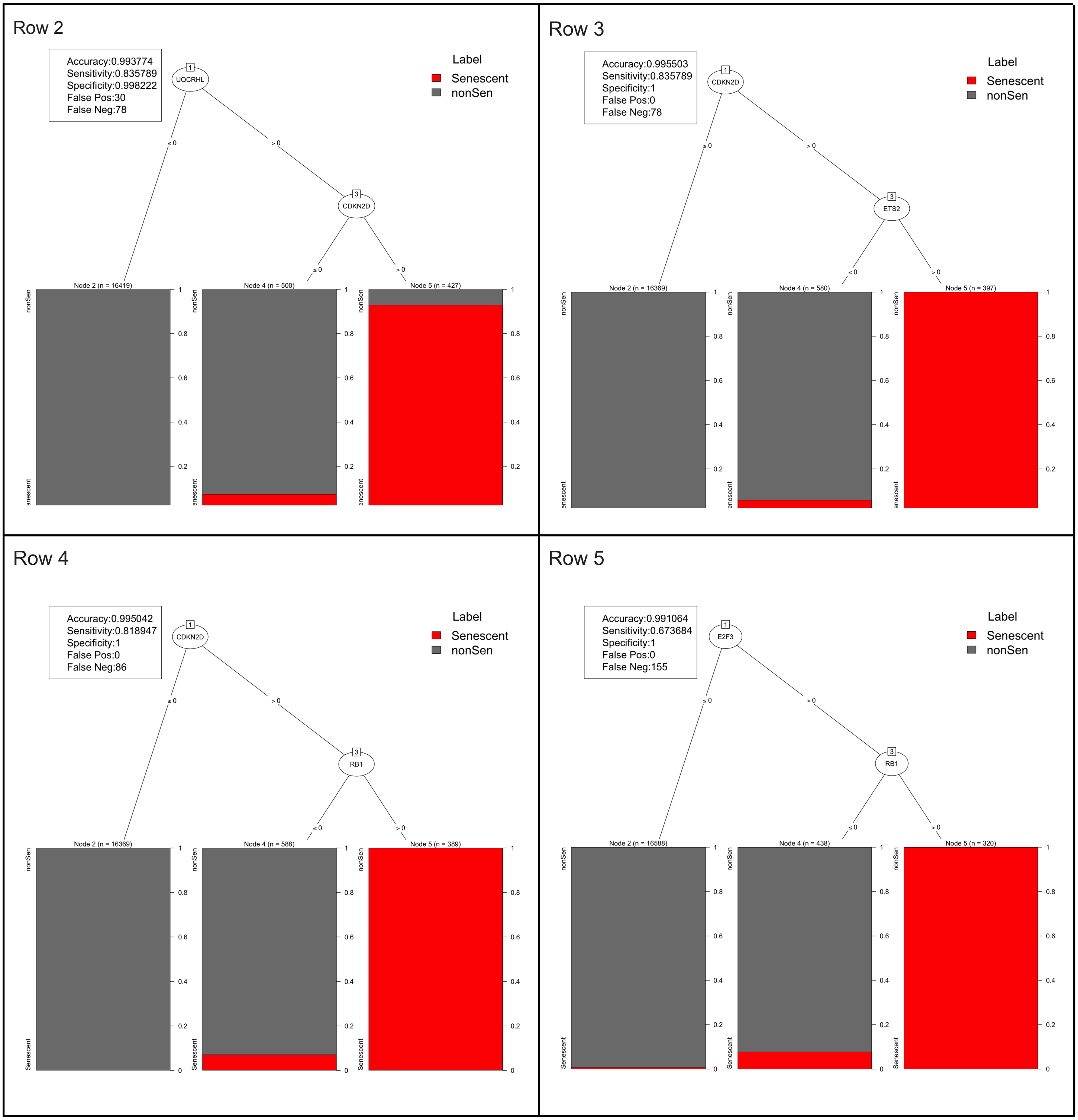

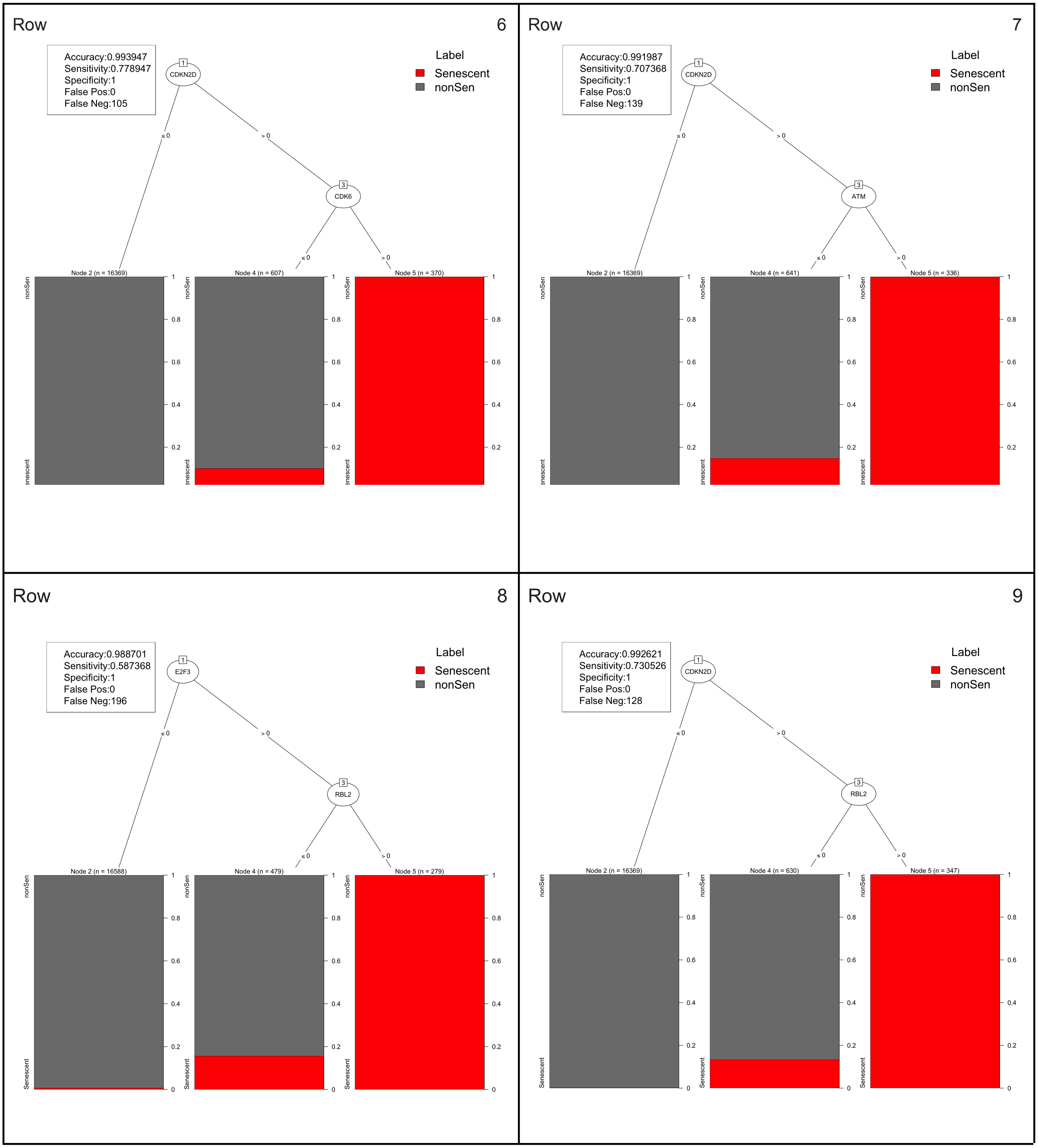

Figure S1. The decision trees correspond to rows of Table 3. All trees are based on binary transformation of Mathys 2019 discovery dataset.

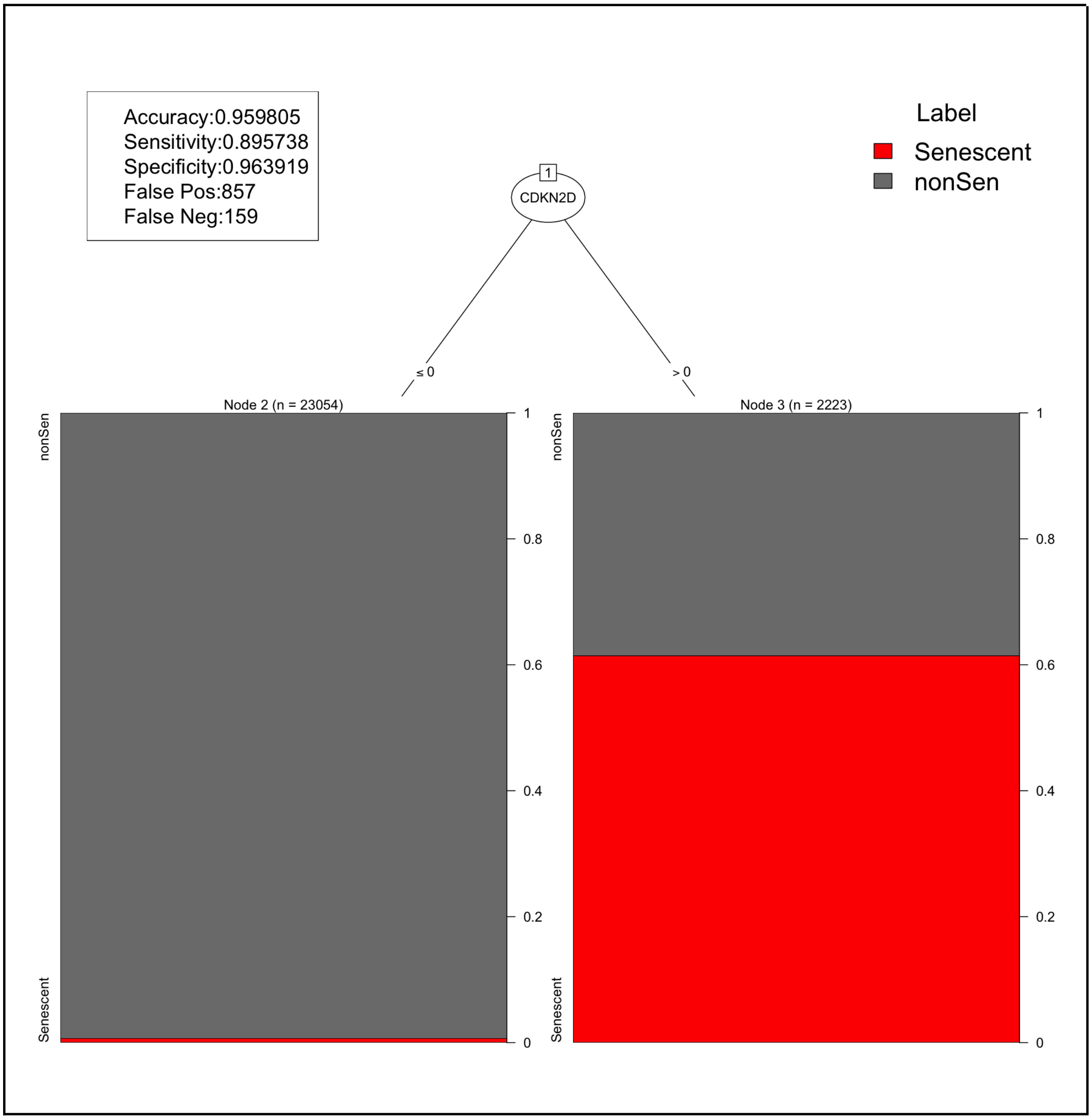

Figure S2. Decision tree trained based on one gene list (CSP) and one marker only, led to significantly lower sensitivity and specificity.

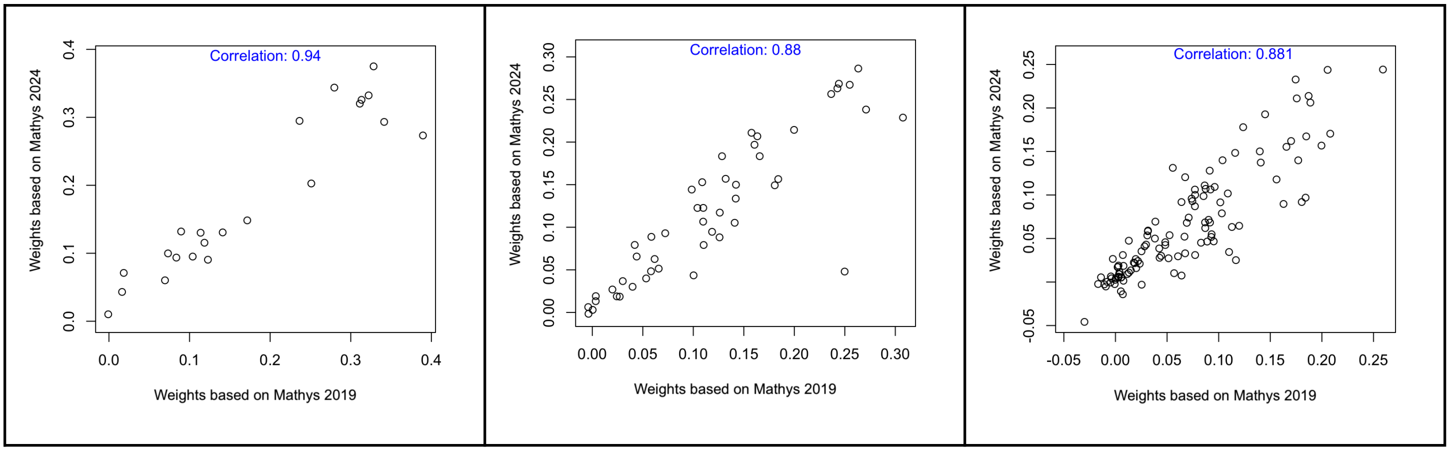

Figure S3. Concordance of eigengene weights between Mathys 2019 and Mathys 2024 discovery datasets. Each panel shows scatter plots comparing gene weights derived from the Mathys 2019 dataset (x-axis) versus those derived from the Mathys 2024 dataset (y-axis), calculated independently for each of the three eigengenes: CSP (left), SIP (middle), and SenMayo (right)
